# Bumetanide Prevents Brain Trauma-Induced Depressive-Like Behavior

**DOI:** 10.3389/fnmol.2019.00012

**Published:** 2019-02-05

**Authors:** Emmanuelle Goubert, Marc Altvater, Marie-Noelle Rovira, Ilgam Khalilov, Morgane Mazzarino, Anne Sebastiani, Michael K. E. Schaefer, Claudio Rivera, Christophe Pellegrino

**Affiliations:** ^1^INSERM, Institute of Mediterranean Neurobiology, Aix-Marseille University, Marseille, France; ^2^Department of Anesthesiology and Research Center Translational Neurosciences, University Medical Center of the Johannes Gutenberg University Mainz, Mainz, Germany; ^3^Laboratory of Neurobiology, Kazan Federal University, Kazan, Russia; ^4^Neuroscience Center, University of Helsinki, Helsinki, Finland

**Keywords:** psychiatric disease, depression, potassium chloride cotransporter 2 (KCC2), bumetanide, neurogenesis, interneuron cell death

## Abstract

Brain trauma triggers a cascade of deleterious events leading to enhanced incidence of drug resistant epilepsies, depression, and cognitive dysfunctions. The underlying mechanisms leading to these alterations are poorly understood and treatment that attenuates those sequels are not available. Using controlled-cortical impact as an experimental model of brain trauma in adult mice, we found a strong suppressive effect of the sodium-potassium-chloride importer (NKCC1) specific antagonist bumetanide on the appearance of depressive-like behavior. We demonstrate that this alteration in behavior is associated with an impairment of post-traumatic secondary neurogenesis within the dentate gyrus of the hippocampus. The mechanism mediating the effect of bumetanide involves early transient changes in the expression of chloride regulatory proteins and qualitative changes in GABA(A) mediated transmission from hyperpolarizing to depolarizing after brain trauma. This work opens new perspectives in the early treatment of human post-traumatic induced depression. Our results strongly suggest that bumetanide might constitute an efficient prophylactic treatment to reduce neurological and psychiatric consequences of brain trauma.

## Introduction

Brain trauma is the main cause of disability all over the world with a very high prevalence in developed countries (Meyer et al., 2008; Bondi et al., 2015). According to the World Health Organization and the Centers for Disease Control and Prevention (Meyer et al., 2008), brain trauma classification is based on multiple factors such as altered neurological functions, brain area of interest and genetic variations. Altogether, these factors lead to highly individualized injuries. Sequels of trauma include low prevalence post-traumatic epilepsies (PTEs), with a severity and occurrence dependent on trauma severity (Kelly et al., 2015; Bragin et al., 2016), and cognitive dysfunctions and depression-like phenotypes are also commonly associated (Peeters et al., 2015; Perry et al., 2015; Stein et al., 2015). Following brain trauma, neuronal cell death occurs and more particularly within the neurons of the dentate gyrus of the hippocampus (Ren et al., 2015; Samuels et al., 2015), leading to hippocampal volume reduction (Samuels et al., 2015; Anacker and Hen, 2017). These observations could be related to changes in post-traumatic neurogenesis in the hippocampus. This has been proposed to be a useful marker of therapeutic treatment efficacy (Brandon and McKay, 2015; Alvarez et al., 2016).

In a wide range of neurological and psychiatric disorders, GABAergic signaling is affected through chloride homeostasis impairment triggered by a down regulation of the main neuronal-specific chloride and potassium extruder, KCC2, and up regulation of the chloride importer NKCC1, respectively (Medina et al., 2014). Similar changes in GABAergic transmission have been reported in a different model of brain trauma (Ben-Ari, 2017). This leads to depolarization and also an excitatory action of GABA that could perturb the generation of behaviorally relevant oscillations and integrative properties of brain networks (Rivera et al., 1999; Luscher et al., 2011; Kahle et al., 2013; Medina et al., 2014; Ben-Ari, 2017). These shifts have been observed notably in developmental disorders including autism spectrum disorders (ASDs) (Tyzio et al., 2014), stroke (Jaenisch et al., 2010; Xu et al., 2016) and epilepsy (Pallud et al., 2014; Tyzio et al., 2014; Kelley et al., 2016). The interaction between major depressive disorders (MDDs) and GABAergic neurotransmission has been suggested in a genetic mice model of GABA(B)-R *knock-out* (Mombereau et al., 2005) and in studies showing an antidepressant effect of potent and selective blockage of GABA(A) transmission (Rudolph and Knoflach, 2011) at both the hippocampus (Boldrini et al., 2013) and mesolimbic system (Kandratavicius et al., 2014). In addition, several observations link chloride homeostasis to secondary neurogenesis through GABA(A) neurotransmission (Luscher et al., 2011; Ostroumov et al., 2016). The generation of new neurons within the DG requires different steps: first, the transition from quiescent to proliferative progenitors, then their differentiation to immature neurons in a GABAergic-dependent manner (Chell and Frisén, 2012; Moss and Toni, 2013). In that context, it’s well-accepted that brain trauma alters neurogenesis (Perry et al., 2015; Stein et al., 2015). In the past decade, the relationship between GABA neurotransmission and neurogenesis has been well-established. Ge and collaborators have shown that GABA receptors are expressed in the progenitor cells and that GABA itself, either ambient or synaptically-released GABA, could act at different steps during neurogenesis from proliferation to cell differentiation and finally synaptic integration (Ge et al., 2006; Anacker and Hen, 2017). In addition, the GABAergic polarity acts on the cell integration (Ge et al., 2006) but also in cell proliferation (Sun et al., 2012), thus establishing a causal link between cell cycling and cell cycle exit on depolarizing GABA condition (Scharfman and Bernstein, 2015; Hu J.J. et al., 2017). Apart from the monoamine hypothesis, a new theory based on the GABA release itself has been proposed to contribute to depression. GABA release has been demonstrated to be impaired in psychiatric disorders and particularly in depression (Luscher et al., 2011; Gabbay et al., 2012). More particularly, the GABAergic receptors have been shown to be decreased in expression and function in the dentate gyrus of depressed patients (Luscher et al., 2011; Lüscher and Fuchs, 2015) and brain tissues collected from suicide patients with a history of depression and anxiety (Merali et al., 2004). One of the first phenomenon linking depression and the hippocampus is the change in hippocampal volume observed both in rodent and in human (Savitz et al., 2010; Schuhmacher et al., 2013; Roddy et al., 2018). This is a common trait observed when the hypothalamic–pituitary–adrenal (HPA) axis is impaired. Other brain regions such as cingulate cortex, prefrontal cortex or even amygdala are also associated with depression (Drevets et al., 2008). In addition to volume changes other functions are changed in the hippocampus of animal displaying DLB, e.g., modified volume (Roddy et al., 2018), impaired GABAergic function (Merali et al., 2004), increase in excitability and monoamine dysfunction (Samuels et al., 2015) as well as impaired secondary neurogenesis and cognitive deficit (Ferguson et al., 2016; Anacker and Hen, 2017). Taken together, this makes the hippocampal formation a important and valuable structure to study depression in TBI models.

Parvalbumin-containing interneurons are the principal source of GABA release within the dentate gyrus and thus potential candidates to explain controlled-cortical impact (CCI)-induced dysregulations through their role in the synchronicity of hippocampal networks (Curia et al., 2008; Drexel et al., 2011; Shiri et al., 2014). Moreover, it is accepted that the activity of this class of interneurons could act on secondary neurogenesis by providing a source of ambient GABA (Song et al., 2012; Butler et al., 2016; Hu D. et al., 2017; Pérez-Domínguez et al., 2017), but little is known about the relationship that exists between parvalbumin-containing interneurons and the establishment of post-traumatic depression (Earnheart et al., 2007; Luscher et al., 2011; Fenton, 2015). Moreover, in human depression, their action is far from being established (Khundakar et al., 2011; Pehrson and Sanchez, 2015; Smiley et al., 2016).

Interestingly, the NKCC1 chloride importer antagonist bumetanide has been shown to attenuate many disorders like ASD, Parkinson’s disease, and schizophrenia as well as some CCI-induced consequences. This stresses the therapeutic potential of restoring low (Cl^-^)_i_ levels and an efficient GABAergic inhibition (Lemonnier et al., 2013, 2016; Damier et al., 2016; Xu et al., 2016; Ben-Ari, 2017). Although, it has been previously shown that bumetanide could have various positive effects on TBI models (Hui et al., 2016; Zhang et al., 2017) and could also act on secondary neurogenesis in stroke condition (Xu et al., 2016), yet nothing is known about the early action of this compound prior to the establishment of depressive-like behaviors (DLB). Our results showed that brain trauma disrupts chloride homeostasis, leading to hippocampal network disturbances and impaired neurogenesis associated with DLB. Early restoration of chloride homeostasis, using the NKCC1 inhibitor bumetanide rapidly after trauma, attenuates the severity of post-traumatic alterations notably by reducing interneuron loss. This, taken together, suggests a therapeutic potential of this FDA-approved compound after trauma.

## Materials and Methods

The French ethical approved all experimental procedures (No. APAFIS#2797-2015112016427629v8). All experiments were performed in blind.

### Controlled-Cortical Impact Model (CCI)

Ten-weeks old C57bl6-J males are housed individually in an enriched environment, consisting in thick rolled paper (Diamon twist, Envigo) and Dome Home (Envigo) allowing correct nesting of the animals as requested by our French ethical committee. They are maintained in a 12 h light/12 h dark cycle environment with controled temperature (23 ± 2°C), food and water are given *ad libitum*. The CCI procedure is performed using aseptic technique. Buprenorphine (0.03 mg/kg) is given intra-peritonealy (i.p.) 30 min before surgery. Anesthesia induction is done using 4% isoflurane mixed with air and enriched with oxygen (0.3%), for the procedure isoflurane is set to 2–2.5%, before animals are positioned in a stereotaxic frame (David Kopf Instruments). Body temperature is maintained at 37 ± 2°C with a heating pad (Harvard Apparatus). The impact is done on the right cortex within the boundaries of the bregma and lambda after a craniotomy is done, using a leica impactor (tip diameter 3 mm, 6 m/s speed, 1.5 mm depth and 200 ms duration). Sham animals receive complete surgery without the impact. Before experiment, animals were randomly assigned to each group, e.g., sham-vehicle, sham-bumetanide (as there were no differences in all considered tests in between sham-vehicle and sham-bumetanide, and to clarify the message of the manuscript, this group will not be presented in the figures), CCI-vehicle and CCI-bumetanide.

### Drug Delivery

Bumetanide stock solution 20 mM (Sigma-aldrich, B3023) is prepared by dissolving 36.4 mg of powder in 1 ml absolute ethanol. The injected solution consists of 40 μl of stock solution diluted in 4 ml PBS 1X. A volume of 26.7 μl par gram of Bumetanide is injected intra-peritonealy, twice daily (9 am and 5 pm), thus corresponding to 2 mg/kg. The vehicle solution consists in the same preparation but lacks the bumetanide powder to respect volume and diluent. Imipramine (30 mg/kg) is i.p injected 30 min before testing the animals for depression.

### Western Blot Analysis

Animals were killed by decapitation after deep isoflurane anesthesia. Hippocampi are quickly dissected out, flash-frozen in liquid nitrogen and stored at -80°C until processed. Brain tissue are homogenized in RIPA buffer (50 mM Tris-HCl pH 8; 150 mM NaCl; SDS 0.1%; Deoxycholic Acid 0.5%; 1% Triton X-100), containing complete Protease/Phosphatase Inhibitor Tablet (Thermo Fisher) and loaded with Laemmli 3X loading buffer. The samples are separated in 4–15% SDS-PAGE gel (Criterion gel, Bio-Rad) and transferred to a nitrocellulose membrane (Whatman). After blocking in Tris-buffered saline/0.1% tween/5% bovine serum albumin (BSA), membranes are exposed overnight at 4°C to primary antibody diluted in blocking solution (Tris-buffered saline/0.1% tween/2.5% BSA), anti-NKCC1 (DHSB, 1:2000), KCC2 [non-commercial (Ludwig et al., 2003); 1:5000] and Phospho-Serine 940 (Rockland, 612-415-E15, 1:1000). Secondary antibodies (anti-mouse HRP, #31430, and anti-rabbit HRP, #31460, Thermo Fisher Scientific) are applied for 2 h at room temperature, before a chemiluminescence assay is performed using horseradish peroxidase-conjugated detection. Signals are revealed using ECL-plus Western blotting reagents (ECL-plus kit, Pierce Biotech) on the image analysis software G box (Syngene). Membranes are then stripped using 50 mM DTT/2% SDS in 50 mM Tris-HCl, pH 7.0 for 30 min at 65°C, and blocked again in Tris-buffered saline/0.1% tween/5% BSA and finally probed with anti-α-tubulin (#62204, Life Technologies) or anti-β-tubulin (TUBB3 18020, Biolegend) for normalization. Signal detection and revelation are done following the same procedure as the one for primary antibodies. Quantifications are performed using Gel Plot Analyzer plugin (ImageJ).

### Immunohistochemistry

Mice are transcardially perfused with 4% paraformaldehyde then 60 μm coronal sections are made and stained overnight at 4°C using KCC2 (1:3000; Ludwig et al., 2003), Bromo-deoxy-Uridin (BrdU) (Dako M0744, 1:100), DCX (Abcam, AB18723, 1:1000) and parvalbumin (Sigma P3088, 1:500), The Alexa Fluor-conjugated secondary antibodies (1/500, Invitrogen) used 2 h at room temperature and slices are finally counterstained with Hoechst 33258 (10 μg/mL, Sigma-Aldrich 861405). Images are acquired using a confocal microscope with 10, 20, 40, or 63X objectives. The KCC2 antibody used in this study is a custom-made antibody recognizing both a and b isoforms of KCC2 (Uvarov et al., 2007), the epitope is localized on the N-terminus part of the protein.

### KCC2 Subcellular Localization Analysis

The measure of the distribution of KCC2 fluorescence associated with cytosolic regions, in sham and CCI condition, is performed at high magnification (x63 objective) using the Image J software. Plot Profiles are done using a line scan analysis through ImageJ software. Briefly, the same straight-line is applied from the extracellular compartment to the nucleus. The intensity profile of each point of the line, separated by 0.1 μm, is analyzed and compared between sham and CCI groups using *t*-test.

### BrdU Injections and Neurogenesis Staining

Intra-peritoneal injections of a 1 mg Bromodeoxyuridine solution (BrdU, Sigma, 10 mg/ml) are performed at 6 days and 1-month post brain trauma to label dividing cells in the S-phase. BrdU is dissolved in PBS 1X. Mice received two BrdU injections (9 am and 5 pm), the day before brain collection. Immunohistochemistry is done using a mouse-BrdU antibody (M0744, 1:100, Dako) to monitor dividing cells and using double cortin (DCX) antibody to label immature newly born granular cells. The total number of either BrdU- or DCX-immunopositive cells are assessed within the granular layer of the dentate gyrus (DG) after images acquisition using an apotome module at 20X objective (BX 40 Olympus). Pictures consist in 1 μm stack images, the total number of stacks gives the total volume of the DG. The total number of positive cells are expressed within the reconstructed volume and reported to the volume, in order to avoid any biases due to thickness differences.

### Behavioral Studies

Animals are habituated to the testing room 1 h before testing. For the open field test (OFT), mice are allowed to freely explore the arena for 10 min (Noldus apparatus, 38.5 cm × 38.5 cm). Parameters are detected and analyzed using the Ethovision software (Noldus).

The forced swim test (FST) paradigm is performed in a 25°C water with first a 2-min habituation period followed by a 4 min recording. The time of immobility is quantified to discriminate between swimming and non-swimming movements. Stabilization movements are not counted as swimming movements.

The tail suspension test (TST) is performed on a 6-min trial and the time of immobility is again measured by the experimenter to discriminate between movements and swinging movements. The splash test consists of spraying a 10%-sucrose solution to the fur of the animal, and then animals are video-monitored for a 5-min period, during which latency to first complete sequence of grooming and total grooming time is measured.

For the novel object recognition test, animals are exposed to an empty open field arena (38.5 cm × 38.5 cm × 38.5 cm) for a 3-min habituation time. In a second time, animals are exposed, in the same arena, to two identical objects for a 3-min period. Finally, after a 1-h delay, animals return to the arena, for a third 3-min period, where one of the objects has been replaced by a new one. The time spent close to the objects is measured and plotted as a new versus familiar object ratio.

### Acute Slices Preparation

Animals are collected on the first post-traumatic week. After cervical dislocation, brains are rapidly removed, the hippocampi dissected, and transverse 350 to 450 μm thickness slices are produced using a Leica VT1000S tissue slicer (Leica VTS1200S, Germany) in oxygenated (95% O_2_ and 5% CO_2_) modified artificial cerebrospinal fluid (mACSF) containing, in mM: 132 choline, 2.5 KCl, 1.25 NaH_2_PO_4_, 25 NaHCO_3_, 7 MgCl_2_, 0.5 CaCl_2_, and 8 D-glucose. Slices are then transferred at room temperature for 1–2 h before chloride and electrophysiological recordings in oxygenated (95% O_2_ and 5% CO_2_) normal artificial CSF (ACSF) containing, in mM: 126 NaCl, 3.5 KCl, 1.2 NaH_2_PO_4_, 26 NaHCO_3_, 1.3 MgCl_2_, 2.0 CaCl_2_, and 10 D-glucose, pH 7.4.

### *In vitro* Electrophysiological Recordings

Hippocampal slices are individually transferred to a recording chamber maintained at 30–32°C and continuously perfused (2 mL/min) with oxygenated normal or adapted ACSF. Extracellular field recordings are made using tungsten wire electrodes (diameter: 50 μm, California Fine Wire, Grover Beach, CA, United States). Recording electrodes are positioned in a pyramidal cell layer of CA3 subfield, and signals are amplified using custom- DAM-8A amplifiers (WPI, GB; low-pass filter: 0.1 Hz; high-pass filter: 3 kHz; gain: x1000) and then acquired using an A/D converter (Digidata 1440A, Axon Instruments). Clampfit 10.1 (Axon Instruments) software is used for the acquisition and analysis of the network activity. Isoguvacine and bumetanide are purchased from Sigma.

### Volumetry Analysis

Coronal 10 μm thick cryostat sections were stained by cresyl violet, digitized and analyzed using an 1.25X objective and computer image analysis system (Optimas 6.51, Optimas Corporation, Bothell, WA, United States). Lesion volume measurement was performed essentially as previously described (Schaible et al., 2014).

### Parvalbumin-Containing Interneurons Quantification

Forty μm sections were stained using a mouse-Parvalbumin antibody (Sigma-Aldrich, 1:500) and counterstained with Hoechst 33258 (Sigma-Aldrich, 10 μg/mL in PBS). The quantification was done in granular layer of the DG at 40X objective. All experiments were manually performed in blind. As done with the BrdU images, pictures consist of 1 μm stack images, the total number of stacks gives the total volume of the DG. The number of positive cells is expressed in percentage within the reconstructed volume to avoid any biases.

### Statistical Analysis

All mean values are given with the standard error mean (SEM). Normality was tested for each distribution and was set to 5%. Two-tailed Student’s, Mann–Whitney test or one-way ANOVA were used accordingly using Prism software (GraphPad Software, Inc., La Jolla, CA, United States). Box plot report the median, the interquartile range and the total range data and represent as following: ^∗^*p* < 0.05; ^∗∗^*p* < 0.01; ^∗∗∗^*p* < 0.001.

## Results

### Behavioral Analysis of Depressive-Like Behavior

**Figure 1:**
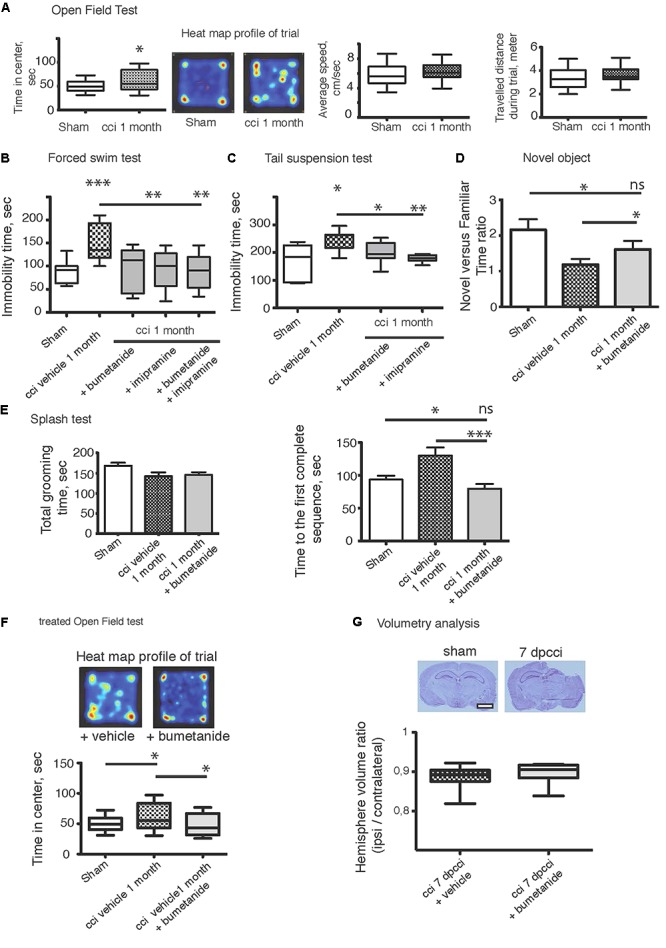
Bumetanide ameliorates CCI induced behavioral changes. **(A)** Open field test (OFT): plots represent both the time spent by the animal in the arena center, the total distance traveled and the average speed of the animal during the 10 min test, sham *n* = 30 and CCI *n* = 20, we used unpaired *t*-test for the comparison on the two population. **(B)** Forced swim test (FST): immobility time in a 25°C water for 4 min, sham *n* = 15, CCI *n* = 12, CCI + bum = 6, CCI + imipramine = 6 and CCI + bum + imipramine = 6. **(C)** Tail suspension test (TST): immobility time for 6 min (sham *n* = 10 and CCI *n* = 10. **(D)** The Novel Objet Recognition shows changes in the exploration time, the results are presented on a ratio of time of new versus familiar, *n* = 16, 15, and 17, respectively. **(E)** Splash test analyzes the total grooming time and the latency to the first complete sequence, *n* = 26, 27, 28, respectively. **(F)** After 1 week of i.p. bumetanide twice daily injection (20 μM). Treated animals showed improvement in the OFT compared to non-treated animals, *n* = 20. Statistic analysis is done using one-way ANOVA test with either Kruskal–Wallis, for non-parametric data or Tukey’s comparison of multiple test for parametric data as *post hoc* treatment to compare between conditions. **(G)** Volumetry analysis: volume are calculated by summation of areas multiplied by distance between sections (500 μm) *n* = 10 brains per condition. The graph shows the ipsi over contralateral volume ratio, 7 days post cci from bumetanide- and vehicle-treated mice. Statistical significance is tested using Mann–Whitney test, *p* = 0.21. ^∗^*p* < 0.05; ^∗∗^*p* < 0.01; ^∗∗∗^*p* < 0.001.

The CCI protocol triggers the appearance of comorbidity factors at later stages, e.g., depression-like behavior. We first performed behavioral tests to ensure that the mice model of brain trauma used in this study exhibited DLB. We performed a FST (Poleszak et al., 2006; Tao et al., 2016), TST (Castagné et al., 2011; Fan et al., 2016), OFT (Tao et al., 2016), splash test (Marrocco et al., 2014; Petit et al., 2014) and finally novel object recognition (García-Pardo et al., 2016; Egeland et al., 2017). All those experiments were carried out 1 month after the CCI (1mpCCI). In the OFT, we observed significant changes in the time spent by the animal in the center of the arena (sham 50.83 ± 13 vs. CCI 60.86 ± 22 s, sham *n* = 30, CCI *n* = 20, *p* = 0.04, Figure 1A) whereas there was no significant difference in the total distance (sham 3308 ± 160 vs. CCI 3571 ± 155 cm, *p* = 0.2, Figure 1A), nor in the average speed of the animals (sham 5.8 ± 0.2 vs. CCI 6.2 ± 0.3 m/s, *p* = 0.3, Figure 1A). Then, we moved to more specific tests for depression using the FST and TST paradigm. We found a significant increase in the immobility time of CCI animals versus sham (sham FST 88.8 ± 6 vs. CCI FST 152.8 ± 12 s, *p* < 0.0001, *n* = 12 and 15 animals respectively; Figure 1B) (sham TST 165 ± 54 vs. CCI TST 246.7 ± 18 s, *p* = 0.007; Figure 1C). To confirm this was indeed a DLB, we injected imipramine (30 mg/kg, *n* = 20 animals), a classical anti-depressant compound given before the test. Interestingly, this compound could be used during behavioral tests, both in an acute and chronic manner. In the acute way, as we used in the study, it is given 30 min before testing animals (Cryan et al., 2005; Castagné et al., 2011; Zhao et al., 2015). In agreement with the literature, we observed a strong effect on the phenotype (CCI FST 152.8 ± 12 vs. CCI imipramine 89.6 ± 30 s for imipramine treated animals, six animals). The same effect on immobility time was observed on the TST (CCI 246.7 ± 18 versus CCI imipramine 168.6 ± 31 s, *n* = 10 per condition) (Figure 1C). Performing a novel object recognition test, a well-known test for MDD (Egeland et al., 2017), we observed a significant change in the time spent by the animal around the new object after CCI, as shown in the new versus familiar time ratio depicted in Figure 1D (sham 2.15 ± 0.2 vs. CCI 1.18 ± 0.15). Finally, after a 10%-sucrose solution was sprayed to the fur, the grooming behavior was assessed (Amini-Khoei et al., 2017). We observed an increase in the time to perform the first entire grooming sequence (sham 93.5 ± 5 vs. CCI 129.9 ± 12 s, *n* = 23 and 24 per condition) without any significant change in the total grooming time (sham 168 ± 7 vs. CCI 142 ± 9 s) (Figure 1E).

### Early Application of Bumetanide Rescues CCI-Induced Depressive-Like Behavior

We then subjected CCI mice to twice-daily i.p. injections of bumetanide (2 mg/kg) during the first week after CCI. The sham and the CCI-vehicle animals received the same procedure with the vehicle solution as described in “Materials and Methods.” This time window was chosen as the blood–brain barrier (BBB) is considered to remain open (Dachir et al., 2014). Behavioral analyses of the cohorts were performed again after 1 month. Analysis of these results revealed a potent action of bumetanide on all the behavioral tests. This indicates a major role of CCI-induced changes in chloride homeostasis in the induction of DLB. The effect of bumetanide was significant in both on the immobility time using FST (*p* = 0.0002, Figure 1B) and the TST (*p* = 0.03, Figure 1C), but also on the exploratory paradigm of the OFT (*p* = 0.04, Figure 1F). The other sets of experiments also showed a beneficial role of bumetanide on the grooming behavior when using the splash test (*p* = 0.0005, Figure 1E) and finally we observed a very potent effect on the novel object recognition paradigm (*p* = 0.0005, Figure 1D). Interestingly, we did not observe any significant difference in the FST paradigm between imipramine-treated animals and bumetanide-imipramine double treated animals (Figure 1B), indicating no additional effect of bumetanide over imipramine.

The accumulation of intracranial pressure is a comorbidity of closed-head TBI and this is produced by the formation of edema. Previous work suggested that changes in chloride homeostasis can have an ameliorating effect on trauma-induced edema (Lu et al., 2007) that could mediate the positive effects of inhibiting changes in chloride transport. Significant formation of intracranial pressure is not expected in this study, as it is an open-head CCI model. To investigate the effect of bumetanide on lesion size, we performed volumetric analysis on both bumetanide- and vehicle-treated CCI brains. By performing cresyl-violet staining, we defined the brain lesion size (Figure 1G). The volume ratio between contralateral and ipsilateral hemispheres showed that, at 7 dpCCI, there was no significant modification in the lesion size after bumetanide application (CCI 0.88 ± 0.01 vs. CCI bumetanide 0.89 ± 0.01, *n* = 10) (Figure 1G).

### CCI-Induced Changes in Hippocampal Network Activity and Inhibitory Strength of GABAergic Signaling

**Figure 2:**
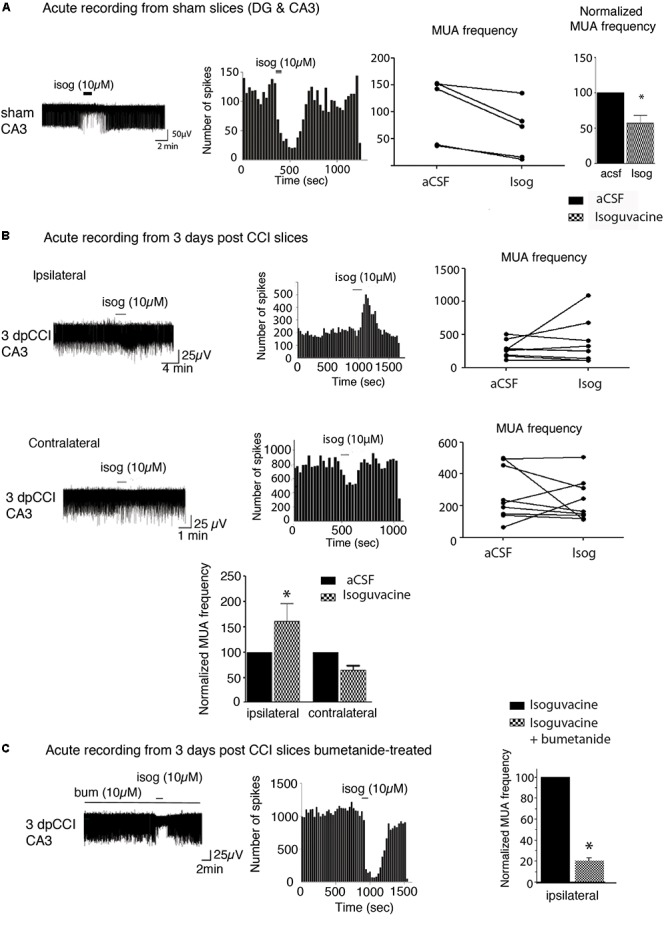
Network activity recording and chloride extrusion efficacy. **(A)** Effect of isoguvacine (10 μM) on hippocampal networks from ipsi and contralateral hippocampus from sham animals. **(B)** Effect of isoguvacine (10 μM) on hippocampal networks at 3 days post-CCI, Top left: example trace of spontaneous extracellular field potentials recorded in ipsilateral hippocampus. Middle: corresponding time course of spike frequency changes. Top right: graph of non-normalized spike frequencies. Middle left: example trace of spontaneous extracellular field potentials recorded in contralateral hippocampus. Middle: corresponding time course of spike frequency changes. Middle right: graph of non-normalized spike frequencies. Bottom: average histograms of normalized spike frequencies. **(C)** The same as in **(B)** with acute pre-treatment of bumetanide (10 μM). 3 days post-CCI (*n* = 2 animals, 4–5 slices per animal). ^∗^*p* < 0.05; ^∗∗^*p* < 0.01; ^∗∗∗^*p* < 0.001.

Studying changes in neuronal activity is important to understand the appearance of MDD. As the hippocampus is one of the main regions known to be involved in the occurrence of DLB, the behavioral tests led us to focus on the hippocampal network activity. The action of bumetanide on the prevention of post-traumatic DLB suggests that changes in chloride homeostasis and in GABAergic neurotransmission in the hippocampus may be involved in the process. Indeed, impairment in chloride homeostasis after TBI has already been shown in the hippocampus in *ex vivo* paradigm (Rivera et al., 2004; Shulga et al., 2012). In order to assess whether GABA(A) transmission was affected in our model, we monitored the effect of GABA(A) receptor activation on extracellular field potentials in the hippocampus. As spontaneous activity of the DG is known to be quite low (Spruston and Johnston, 1992; Kvajo et al., 2011) (Supplementary Figure 1), we decided to record multi-unit activity (MUA) from the CA3 hippocampal region. Acute hippocampal slices, both from ipsi- and contra-lesional hemispheres at 3 days after CCI were recorded in the presence of 10 μM isoguvacine, a potent and selective GABA(A) agonist. Such treatment exerts an excitatory action on the action potential spiking frequency on ipsi- but not on the contralateral hippocampus at 3 dpCCI (Figure 2B), as compared to the sham condition (Figure 2A). This set of results suggests that GABAergic transmission is modified, rendering the network more excitable. The strong block of the depolarizing effect of isoguvacine by bath application of 10 μM bumetanide (*n* = 2 animals, 4 to 5 slices per animal) (Figure 2C) indicates the involvement of chloride imbalance in the CCI-induced changes in GABA(A) responses.

### Changes in Chloride Regulatory Proteins After CCI

We then investigated whether changes in network excitability could be explained by changes in dynamics of chloride extrusion efficacy. To estimate to what extent chloride-regulatory proteins are affected during the early post-traumatic time window, CCI, NKCC1, and KCC2 protein expression levels were followed during the first post-traumatic week in the hippocampus.

**Figure 3:**
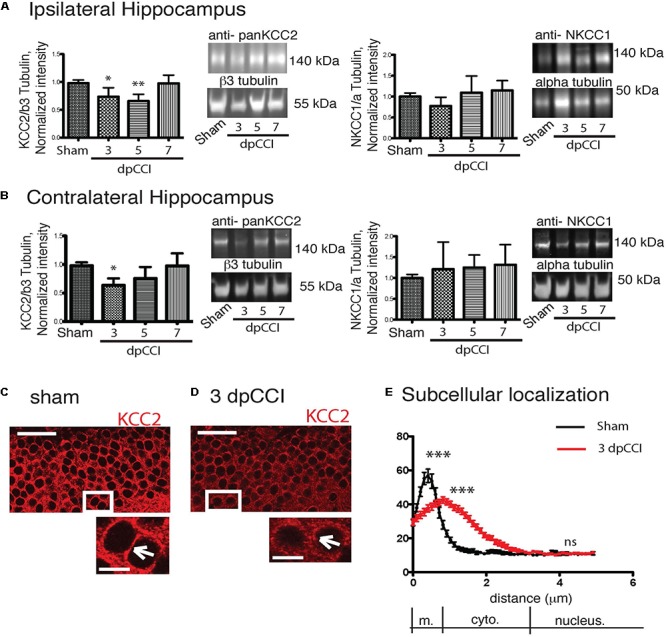
CCI-induced changes in chloride co-transporters expression. **(A)** The left panel represents the KCC2 protein expression normalized to the neuronal marker β3-tubulin on the ipsilateral hippocampus. Protein expression over the time is expressed in comparison to the sham conditions. On the right panel, NKCC1 protein expression is shown normalized to the ubiquitous marker α-tubulin. Protein expression over the time is expressed in comparison to sham conditions, *n* = 8 per condition. One-way ANOVA test is performed and expressed as following ^∗^*p* < 0.05; ^∗∗^*p* < 0.01; ^∗∗∗^*p* < 0.001 together with Kruskal–Wallis *post hoc* test. **(B)** Same as **(A)** but in the contralateral hippocampus. **(C–E)** KCC2 staining in granule cells. **(C)** Sham at 3 dpCCI. The labeling is at the cellular membrane (arrowhead) and the cytoplasm is almost devoid of KCC2 labeling. **(D)** 3 dpCCI. KCC2 is found in the cytoplasmic cell compartments (arrowheads). **(E)** Histograms representing the distribution and quantification of the intensity of fluorescence in 3 dpCCI cells (red curve) in sham (black curve). Statistical analysis represents the difference in each sub region of the cell, namely membrane, cytoplasm, and nuclear staining. Scale bars: 50 and 10 μm.

We observed a significant decrease in KCC2 protein expression rapidly after the trauma with a recovery on the 7^th^ day at the ipsi and contralateral hippocampus (Figure 3A,B). For NKCC1 analysis, we did not observe any significant changes in protein levels in the hippocampi (Figure 3A,B). Similar results were obtained for KCC2 and conversely for NKCC1 at mRNA levels in the injured hemisphere (Supplementary Figure 3).

### CCI-Induced Internalization of KCC2 Plasma Membrane

To study a possible link between protein expression and network activity changes, we then investigated whether KCC2 expression and more specifically its subcellular distribution were affected. We used a specific KCC2 antibody to examine the cellular distribution of KCC2. We decided to focus on DG, a hippocampal region where changes in network activity is already reported after TBI (Bonislawski et al., 2007) and as the DG region is involved in depression. In sham granular cells, KCC2 was mainly located near the membrane of cell bodies (Figure 3C). In contrast, the labeling of KCC2 in granular cells was largely cytoplasmic 3 dpCCI (Figure 3D). The cellular distribution of KCC2 in sham and 3 dpCCI was significantly different with a peak around the membrane for sham granular cells (sham 64.09 ± 4.550 *N* = 2 *n* = 60 vs. 3 dpCCI 44.34 ± 1.83 *N* = 3 *n* = 88), together with staining dispersion over the cytoplasmic compartment in granular cells at 3 dpCCI (sham 13.56 ± 1.011 *N* = 2 *n* = 60 vs. 3 dpCCI 31.92 ± 1.54 *N* = 3 *n* = 88) (Figure 3E). This suggests an internalization of KCC2 after TBI and is in agreement with robust changes in chloride homeostasis and GABAergic transmission in the DG.

To examine if GABAergic transmission is altered all over the hippocampus, we decided to also study the functionality of chloride transport using Clomeleon mice in the CA1 region (Berglund et al., 2006) (Supplementary Figure 2). We found that chloride extrusion was significantly reduced at 3 and 5 but not at 7 dpCCI compared to sham condition, thus confirming the phenotype observed using the MUA approach and the biochemical techniques. Our results suggest a general effect of CCI on chloride transport into the hippocampal formation through the reduction of KCC2 expression and function.

Taken together, these results show an imbalance in the NKCC1/KCC2 ratio in favor of NKCC1 and a loss of function of KCC2.

### Bumetanide Rescues Post-traumatic Impairment in Secondary Neurogenesis

**Figure 4:**
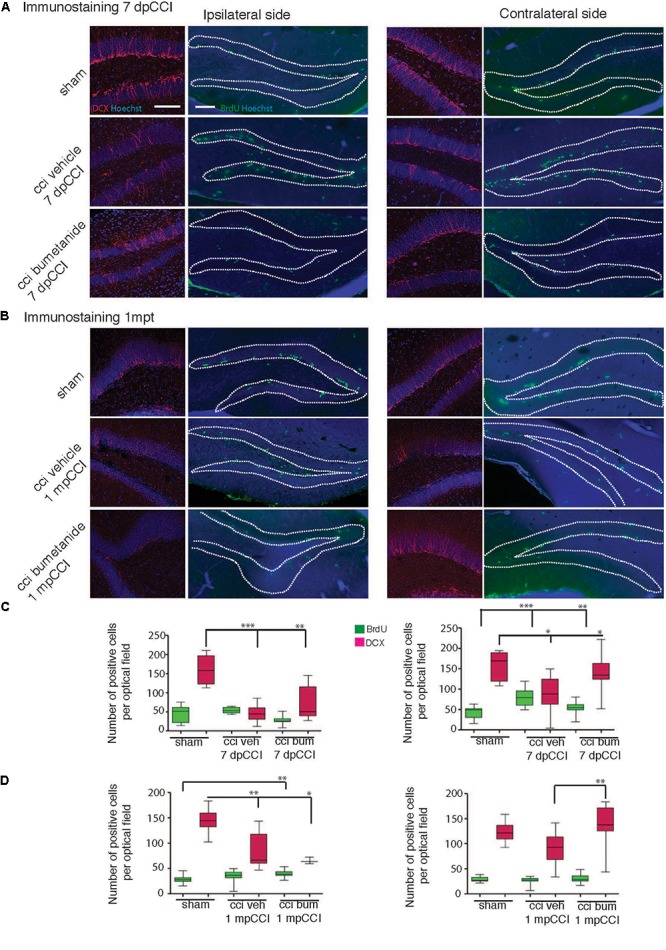
Effect of bumetanide on CCI-induced changes in secondary neurogenesis. Secondary neurogenesis in the dentate gyrus. **(A)** Double-cortin (DCX) and BrdU labeling at 7 days post-CCI in the ipsilateral (left) and contralateral (right) dentate gyrus of sham, CCI vehicle and bumetanide-treated animals. Dotted lines delimit granular layer of dentate gyrus (scale bar = 100 μm). **(B)** Same as in **(A)** at 1 month post-CCI. **(C)** Quantification of BrdU and DCX positive cells 7 dpCCI in the ipsilateral (left) and contralateral (right) dentate gyrus of sham, CCI vehicle and bumetanide-treated animals. **(D)** Same as in **(C)** at 1 month post-CCI. DCX 7 days post-CCI: *n* = 6 animals per condition, 3 slices per animal; 1 month post-CCI; *n* = 4 animals per condition, 2–4 slices per animal. BrdU 7 days post-CCI: *n* = 5 sham *n* = 6 CCI vehicle and 6 CCI bumetanide, 2–6 slices per animal, 1 month post-CCI: *n* = 3 sham *n* = 4 CCI vehicle and 4 CCI bumetanide, 3–4 slices per animal. All sets of data were analyzed using one-way ANOVA test with Tukey’s *post hoc* test. ^∗^*p* < 0.05; ^∗∗^*p* < 0.01; ^∗∗∗^*p* < 0.001.

Previous results suggested that the effect of antidepressants on proliferation of adult born neurons of the DG might be involved in the mechanism of action of these compounds. Considering the prophylactic anti-depressant effect of bumetanide found in this study, it is plausible that part of the antidepressive effect is mediated by changes in proliferation. Thus, we monitored both the proliferative cells and newly born neurons at the end of the first post-traumatic week. Neurons were labeled with double-cortin (DCX), a marker of immature neurons (Ren et al., 2015), and proliferative cells were stained with bromo-deoxy-Uridin (BrdU) to assess the relative number of dividing cells within the granular layer of the DG (Samuels et al., 2015). The number of positive cells was calculated on a defined slice volume (see section “Materials and Methods”) and expressed as the total number of positive cells per volume. We observed a significant CCI-induced reduction in the number of the DCX positive neurons within the DG both in the ipsi- and contra-lesional hippocampi at 7 dpCCI (sham ipsi 168.3 ± 26.8 vs. CCI ipsi 45.9 ± 11.09 and sham contra 158.8 ± 15.3 vs. CCI contra 89 ± 21.8; respectively 6 and 4 animals, 4 slices per animal) (Figure 4A,C), together with an increase in the number of BrdU positive cells within the DG (sham ipsi 45 ± 5.8 vs. CCI ipsi 53.5 ± 4.3 and sham contra 43.5 ± 4.2 vs. CCI contra 81.3 ± 6.7, *n* = 4 and 6 animals, 6 slices per animal) (Figure 4A,C). Bumetanide treatment reduced first the number of BrdU positive cells (CCI bumetanide ipsi 27.9 ± 3.8 and contra 54 ± 3, *n* = 4 animals, 6 slices per animal) (Figure 4A–C) and triggered an increase in the number of newly generated neurons (CCI bumetanide ipsi 69.2 ± 11 and contra 138.4 ± 11, *n* = 4 animals, 4 slices per animal) (Figure 4A–C).

Interestingly, 1 month after trauma, it was not possible to find any significant differences in the number of BrdU+ (sham contra 29 ± 1.3 vs. CCI contra 26 ± 1.6) and in the number of DCX+ neurons (sham contra 124 ± 6.1 vs. CCI contra 91.3 ± 8.5) within the contralateral hippocampus of CCI animals compared to sham animals (Figure 4B–D). On the contrary, a significant and persistent loss of newly generated neurons (sham ipsi 145.3 ± 7.3 vs. CCI ipsi 85.3 ± 11.3) with no significant change in BrdU positive cells (sham ipsi 28.3 ± 1.9 vs. CCI ipsi 34.9 ± 3.9) (Figure 4B,D) was still present at the ipsilateral side, indicating a permanent change at the DCX level compared to the transient one observed in the contralateral side. This suggests that both hemispheres are involved in the early settling up of post-traumatic depression.

**Figure 5:**
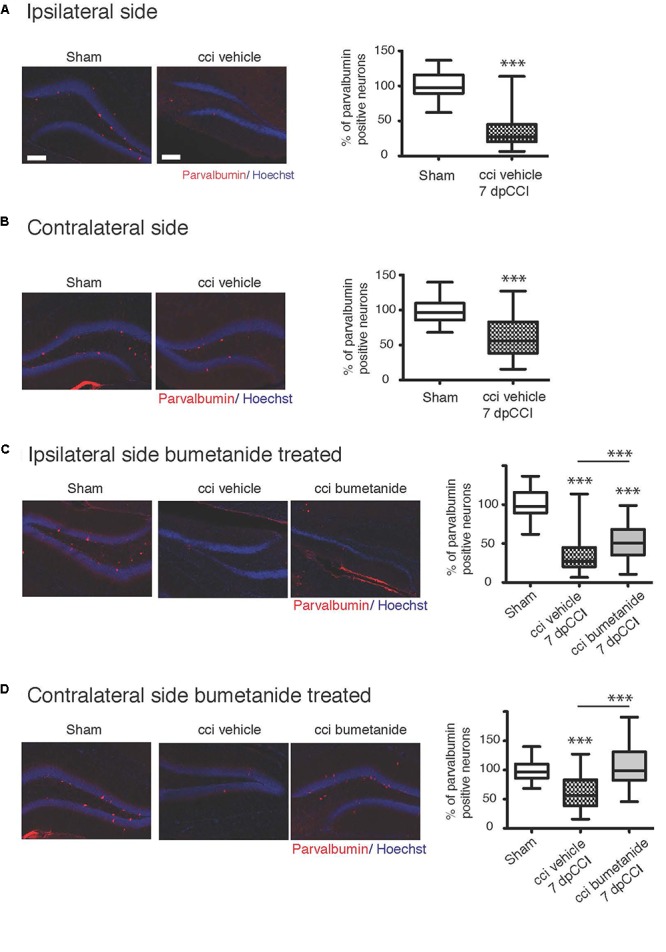
Effect of bumetanide on CCI-induced parvalbumin positive interneuron death. **(A)** Ipsilateral hippocampus: left panel example of parvalbumin and Hoechst immunostaining, from sham and CCI mice. On the right panel, quantification of parvalbumin positive interneurons in the dentate gyrus normalized to sham values. *n* = 5 animals per condition. **(B)** Same as **(A)** but in contralateral hippocampus, the histogram shows reduction in the number of parvalbumin-containing cells in the DG, *n* = 5 animals per condition. **(C)** Effect of bumetanide in parvalbumin interneuron survival in the ipsilateral hippocampus. The histogram shows a significant reduction in the cell loss in the presence of bumetanide but this is though significantly less as compared to sham, *n* = 5 animals per condition. **(D)** Contralateral hippocampus: bumetanide injection reduces interneurons loss, *n* = 5 animals per condition. All sets of data were analyzed using one-way ANOVA test with Tukey’s *post hoc* test. One-way ANOVA test is expressed as following ^∗^*p* < 0.05; ^∗∗^*p* < 0.01; ^∗∗∗^*p* < 0.001.

Ambient GABA, that is provided by the activity of DG interneurons, may play a role in the proliferation and migration of granular cell progenitors (Duan et al., 2008). We then wonder if GABAergic signaling known as a neurogenesis modulator (Moss and Toni, 2013; Samuels et al., 2015; Alvarez et al., 2016) could contribute to the etiology of post-traumatic depression (Cryan et al., 2005). Therefore, it appeared interesting to quantify parvalbumin interneurons, which are known to play a critical role in post-traumatic consequences (Drexel et al., 2014; Hsieh et al., 2014; Khodaie et al., 2014). We quantified the parvalbumin-containing interneurons survival in the granular layer of the DG, both from ipsi and contralesional hippocampi at 7 dpCCI. Both sides showed a significant reduction of the number of parvalbumin-positive interneurons, compared to sham condition (ipsi 67 ± 4.6%, ^∗∗∗^, *n* = 5 animals, 25 slices; contra 40 ± 6%, ^∗∗∗^, *n* = 5 animals, 26 slices, Figure 5A,B). This loss was significantly reduced by bumetanide application at the contralateral (101 ± 5.3%, ^∗∗∗^, *n* = 5 animals, 3 to 4 slices per animal, Figure 5D) and ipsilateral side (52 ± 3.9%, ^∗∗^, *n* = 5 animals, 3 to 4 slices per animal, Figure 5C), compared to the CCI condition itself. Thus, the effect of bumetanide on DG secondary neurogenesis could be partly caused by changes in ambient GABA that is provided by the activity of DG interneurons.

## Discussion

**Figure 6:**
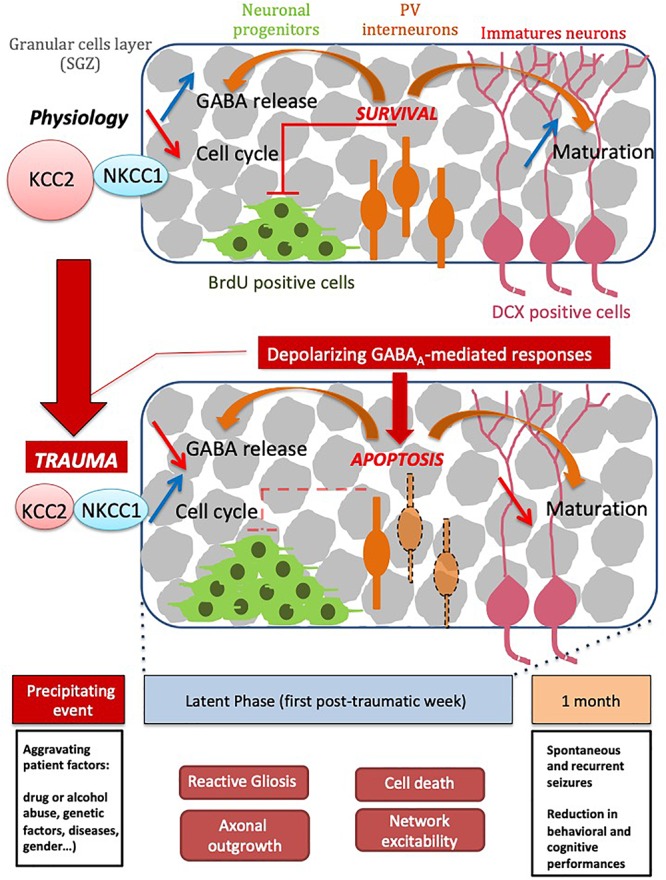
Schematic scheme of TBI time course events. Please note that series of events taking place right and shortly after trauma are sequentially arranged leading to both short and long term consequences leading to decreased cognitive performance.

Our fundamental issue was to understand the consequences of the depolarizing GABA at both early and late stages after brain trauma. Using our experimental model, we found that CCI-induced DLB is strongly sensitive to trauma-induced changes in GABA(A)-mediated responses. The depolarizing GABA(A) responses, at very early stages after CCI, lead to DLB phenotypes at chronic stages. The question raised by these results is how changes in GABA(A) transmission are involved in the CCI-induced rearrangements in the hippocampal network, leading to abnormal behavior (Figure 6). Our analyses after CCI highlight long-term impairment of mood-associated behavior. CCI mice exhibited a phenotype that mimicks a decreased defensive behavior in an unfamiliar environment, as it has been described in other anxiety-like behavioral tests (Pandey et al., 2009; Stemper et al., 2015). Surprisingly, blocking GABA(A)-mediated depolarization, with the specific inhibitor bumetanide at early stages after CCI, resulted in a significant long-term reduction in DLB, long after the end of the treatment with bumetanide. These results pinpoint an important role of the qualitative changes in GABA(A) responses and suggest that bumetanide itself could act in a prophylactic manner as an anti-depressant compound; this was proven to be independent of its effect on lesion volume in a model of cerebral ischemia (Xu et al., 2016) and in our hands. Consistent changes in KCC2 and NKCC1 expression have been found in a number of trauma models, as well as in resections form temporal lobe epilepsy (Pallud et al., 2014). Qualitative changes in GABA(A) transmission and levels of chloride regulatory proteins have not however been well-characterized in CCI models (Robel et al., 2015; Hui et al., 2016). In the present study, we show that KCC2 expression is significantly changed in the hippocampus during the first week following trauma. Interestingly, although these changes occur shortly after CCI in both the ipsi and contralesional hippocampi, they remain transient as KCC2 expression levels return to normal values in both hippocampi. Changes in chloride extrusion efficacy are consistent with this biochemical conclusion, thus resulting in a switch in GABA(A)-mediated network excitability as opposed to the control condition. As expected, the effects observed in the contralateral hippocampus are milder but present. This put the question to how these transient changes could have a long-term effect on brain circuitry. Previous results showed that almost all antidepressants have significant effects on proliferation in the DG and production of newborn neurons. This has led to the hypothesis that proliferation in the DG is associated with DLB. While CCI induced a significant increase in proliferation in the contralateral DG alone, the number of DCX positive immature neurons was significantly diminished on both sides. The acute effect of bumetanide that we observe, leads to increased neuron production and reduced proliferation. The results we show here propose that the neurogenesis itself is modified leading to a reduction in neuron production. The remaining question is how this transient KCCs effect could last longer. Our results provide evidences that transition from BrdU positive cells to double-cortin positive immature neurons after CCI is significantly affected by blocking GABA(A)-mediated responses at early stages. This, together with the interneurons cell death, leads us to propose that GABAergic neurotransmission, either qualitative and quantitative, impacts the secondary neurogenesis (Scharfman and McCloskey, 2009; Toda and Gage, 2017). Further studies are now needed to discriminate the exact role of chloride transporters in both cell cycle and cell differentiation.

Other TBI features must be taken into account to explain the action of bumetanide seen in this study. Among them, inflammation is the most common phenomenon happening after brain insults (Solmaz et al., 2009; Anderson, 2013; Anderson et al., 2014). This inflammation could have several effects, among which, cell-death is one of the most prominent effects. It has been shown in other models that inflammation is associated with a massive loss of hippocampal formation write in epilepsy (Aldenkamp and Bodde, 2005; Swartz et al., 2006; Jefferys, 2010; Peng et al., 2013) or TBI models (Swartz et al., 2006; Morrison et al., 2011; Belousov et al., 2012; Acosta et al., 2013) and more particularly in the DG region (Lowenstein et al., 1992; Kourdougli et al., 2015; Sun et al., 2015). In that context, neuronal cell death and more specifically Parvalbumin positive cell death is one particular phenomenon both in epilepsy (Huusko et al., 2015) and in TBI models (Santhakumar et al., 2001; Cantu et al., 2014; Huusko and Pitkänen, 2014). In those studies, even with a milder model, they were able to show a really strong effect in Parvalbumin cell survival. Although it’s known that principal cells required KCC2 for their survival (Lee et al., 2007; Pellegrino et al., 2011), nothing is known about interneurons survival in that particular context. One explanation could be that interneurons are not dying but lose their biochemical identity after trauma since this has been demonstrated in other trauma models (Unal-Cevik et al., 2004; Todkar et al., 2012; Kelley et al., 2016). In our model, interneurons also lose their GAD67 immunoreactivity but this loss is not transient as shown in other models (Kelley et al., 2016) since it persists after 1 month, thus, confirming a significant and permanent reduction in the number of parvalbumin-positive interneurons. Disruption of the BBB is also an important in leading to cell death and brain invasion. In our model the bbb remains open during the first post-traumatic week, thus the crossing of the BBB is possible during that specific time period (Dachir et al., 2014). In other studies, its disruption was observed (Cernak, 2005; Davidsson and Risling, 2011; Zhu et al., 2018) and proposed to be linked to glial and microglial invasion. Again, further studies are necessary to determine the exact contribution of chloride homeostasis in inflammation. One other brain trauma feature we need to add to this discussion is the cell volume regulation and more particularly, the neuronal volume regulation. It has been proposed that, after brain insults, neurons could have volume variation in an aquaporin-independent process (Hoffmann et al., 2009; Zeuthen, 2010; Ullah et al., 2015). This aspect needs to be linked with the structural plasticity observed in the principal cells after trauma in which both the dendritic tree (Winston et al., 2013; Wang et al., 2016) and dendritic spines are affected (Swann et al., 2000). One possibility could be that bumetanide, through its action on hyperexcitability, could prevent protein remodeling observed after TBI, thus preventing volume changes. One of the questions of interest in our case regards bumetanide action and the so-called therapeutic window we used. We decided to work on the first post-traumatic week due to the poor BBB permeability for bumetanide (Töllner et al., 2014), and as BBB is known to be opened at that stage (Dachir et al., 2014). Even if the peripheral action of bumetanide could have effect in the CNS, we consider our main effect to be central as shown by the effect on parvalbumin survival and on neurogenesis.

Although the CCI-induced changes in chloride regulatory proteins and GABA(A) transmission are consistent with short-term effects on excitability, it is less obvious how this is involved in a long-term effect after trauma. A possible contributing explanation could be related to cell death of interneurons. We have previously shown that the qualitative changes in GABA(A) responses is tightly linked with the survival mechanism of injured neurons (Pellegrino et al., 2011; Shulga et al., 2012). Changes in the interneuron population leading to changes in GABA release could significantly change the excitability of the network (Hsieh et al., 2014; Shiri et al., 2014). In some pathological contexts, such as temporal lobe epilepsy and TBI as well as in another different model of acquired epilepsy, parvalbumin interneurons are known to be very sensitive to death (Drexel et al., 2011; Hsieh et al., 2014) and their loss is involved in the dentate gyrus hyperexcitability triggered by aberrant sprouting (Zhang and Buckmaster, 2009). Altogether this highlights that both can lead to changes GABA polarity at early stages and impaired GABAergic signaling. We have proposed that bumetanide could prevent trauma induced cell death (Shulga and Rivera, 2013; Hui et al., 2016). This mechanism involved the block of the post-traumatic depolarizing effect of GABA(A) receptor that is produced by KCC2 functional downregulation (Lee et al., 2011; Pellegrino et al., 2011; Winkelmann et al., 2015; Hui et al., 2016). The results presented here clearly show that bumetanide prevents CCI-induced interneuron death at least in the DG. Thus, the previously shown mechanism for trauma-triggered apoptosis of principal cells could also be relevant for interneurons. The activity of parvalbumin interneurons has been linked to changes in secondary neurogenesis in the DG. Released ambient GABA from hilar interneurons, and this is of primary importance for regulating the proliferation state of cells within the DG (Boldrini et al., 2013; Moss and Toni, 2013; Samuels et al., 2015; Alvarez et al., 2016). Thus, the effect of bumetanide on interneuron survival could contribute to the short and long-term effects on DG proliferation (Sun et al., 2012).

In the present study we have mainly focused on DLB but bumetanide amelioration of other pathological behaviors may be also present. It will be highly interesting to supplement these studies with other cognitive tests for, e.g., learning and memory together with social interaction paradigms. We show that each hemisphere reacts to the brain trauma to different extents and with different kinetics. This highlights that the consequences in the contralateral hemisphere are as important as in the ipsilateral side (Khalilov et al., 2003), and thus cannot be considered as an independent structure in the etiology of DLB. This result also prompts testing of the specific compound to block NKCC1 able to penetrate the BBB or promote KCC2 function after CCI to restore GABAergic activity (Gagnon et al., 2013; Medina et al., 2014; Puskarjov et al., 2014).

## Conclusion

Our study pinpoints the contribution of the depolarizing GABA in the establishment of the TBI-induced DLB. This work opens new perspectives to treat TBI-associated psychiatric disorders and suggests the use of bumetanide as a potential prophylactic agent.

## Author Contributions

EG was responsible for biochemistry, IHC, TBI model, electrophysiology, and surgery. MA performed the volumetric analysis and IHC. MNR performed the behavioral analysis. MM performed the behavioral analysis and IHC. IK performed the multi-unit recording. AS performed the neuroscoring and surgery. MS and CR designed the experiment and wrote the manuscript. CP performed the behavioral analysis, designed the experiment, wrote the manuscript.

## Conflict of Interest Statement

The authors declare that the research was conducted in the absence of any commercial or financial relationships that could be construed as a potential conflict of interest.
