# Supplementary material for: Bumetanide Prevents Brain Trauma-Induced Depressive-Like Behavior
Source: Front Mol Neurosci. 2019 Feb 5;12:12. doi: 10.3389/fnmol.2019.00012 (PMC6370740; doi:10.3389/fnmol.2019.00012)
Supplement: Supplementary file 1 [file Data_Sheet_1.PDF]

A) aCSF MUA frequency: DG versus CA3 region

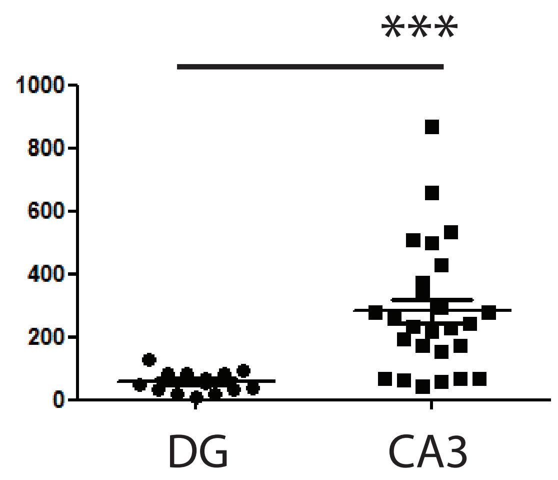


B) 3dpCCI

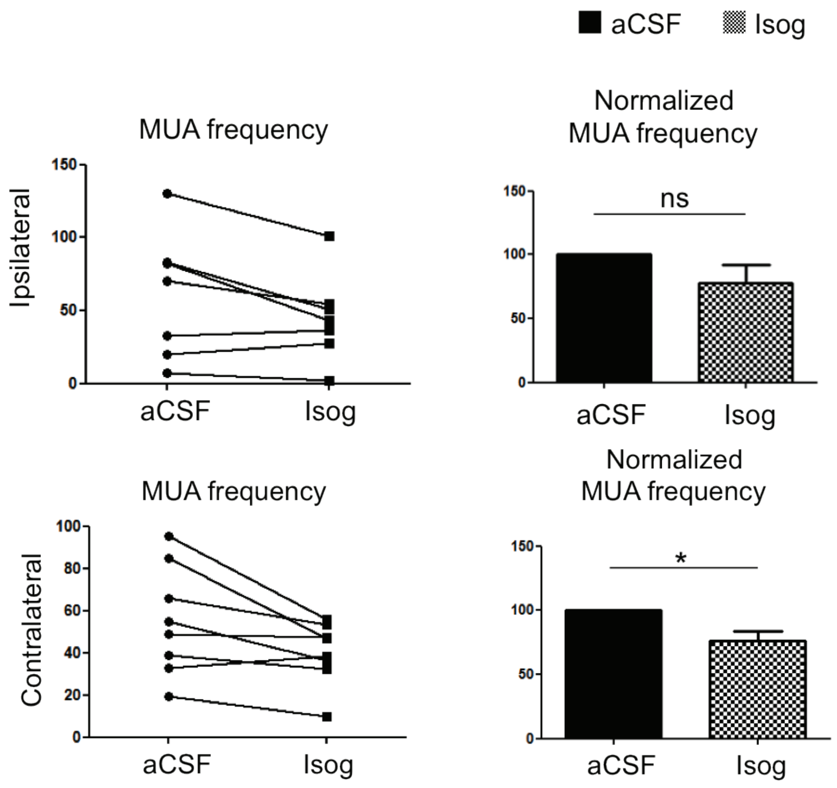


A) Summary of the recorded activity on the DG versus CA3 on our P16 weeks old animals. there is a large and strong activity in CA3 slices whereas the activity in the DG is poor over the recordings. Experiment coming from 4 to 5 different animals 3 to 6 slices recorded per animals.

B) Here, is a comparative description of the effect of isoguvacine applied on the hippocampal slices. There is a the differential effect in the responses seen in the ipsilateral versus the contralateral dentate gyrus. Already at 3 dpCCI isoguvacine does not produce any significant change in the slice multi unit frequencies whereas in the contralateral side, isoguvacine is still hyperpolarizing. This highlight the difference in MUA frequency that already exists after 3 days in the traumatized hippocampus and the progressive changes occuring within the structure. Experiment coming from 4 different animals 3 to 6 slices recorded per animals.
