# Supplementary material for: Bumetanide Prevents Brain Trauma-Induced Depressive-Like Behavior
Source: Front Mol Neurosci. 2019 Feb 5;12:12. doi: 10.3389/fnmol.2019.00012 (PMC6370740; doi:10.3389/fnmol.2019.00012)
Supplement: Supplementary file 2 [file Data_Sheet_2.PDF]

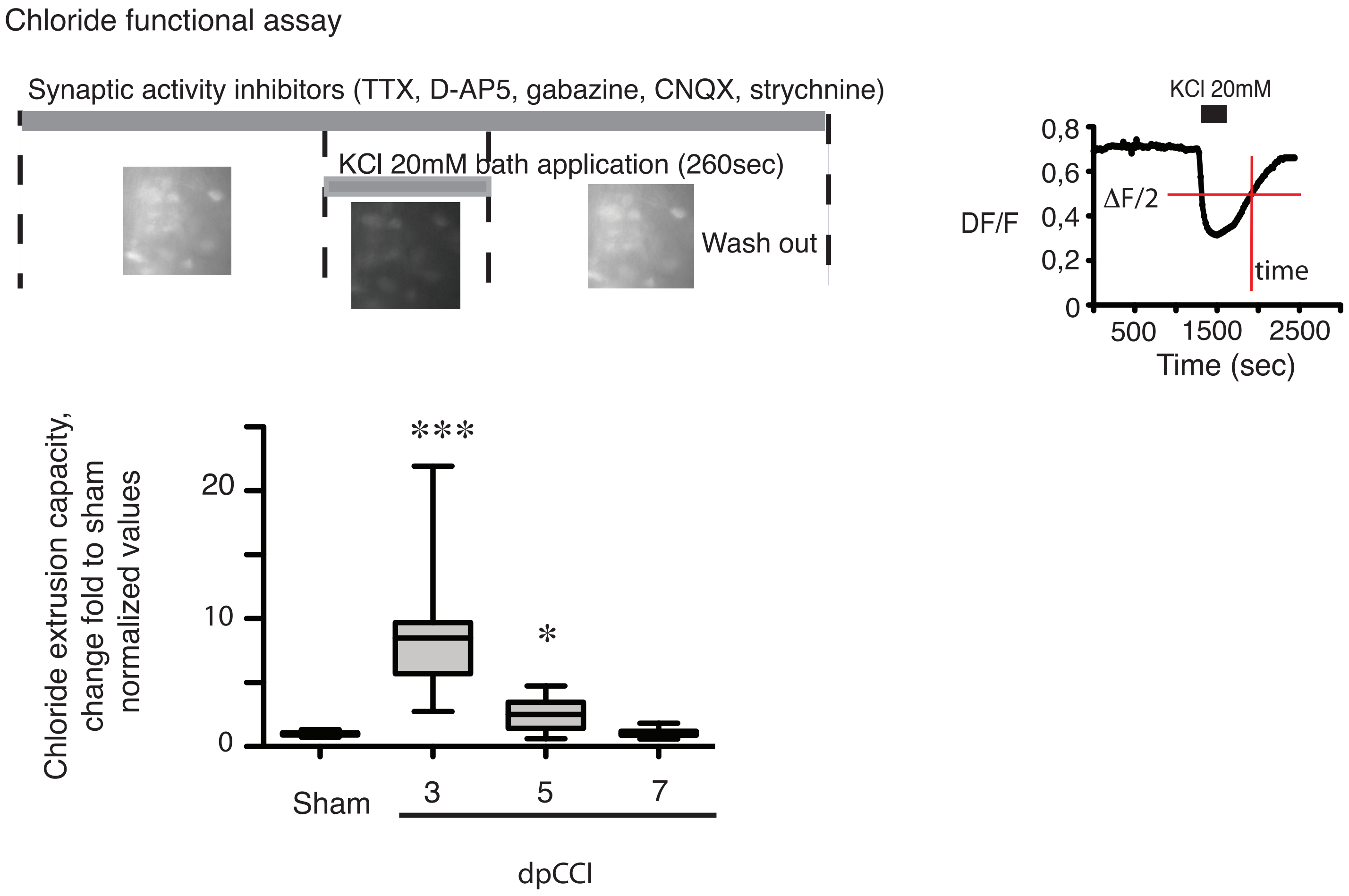

Experimental design of the chloride recording: Bath application of 25mM potassium chloride solution for 260sec in presence of all synaptic blockers. Both the amplitude of the response triggered by the bath application and the recovery of the signal is quantified. Results are represented as chloride extrusion capacity by reporting the fluorescence recovery, meaning the extrusion capacity depending on the amplitude of the signal, to avoid any bias due to changes in loading amplitude.

Goubert et al., Supplemental figure 2
