# Supplementary material for: Bumetanide Prevents Brain Trauma-Induced Depressive-Like Behavior
Source: Front Mol Neurosci. 2019 Feb 5;12:12. doi: 10.3389/fnmol.2019.00012 (PMC6370740; doi:10.3389/fnmol.2019.00012)
Supplement: Supplementary file 3 [file Data_Sheet_3.PDF]

A) Quantitative PCR analysis

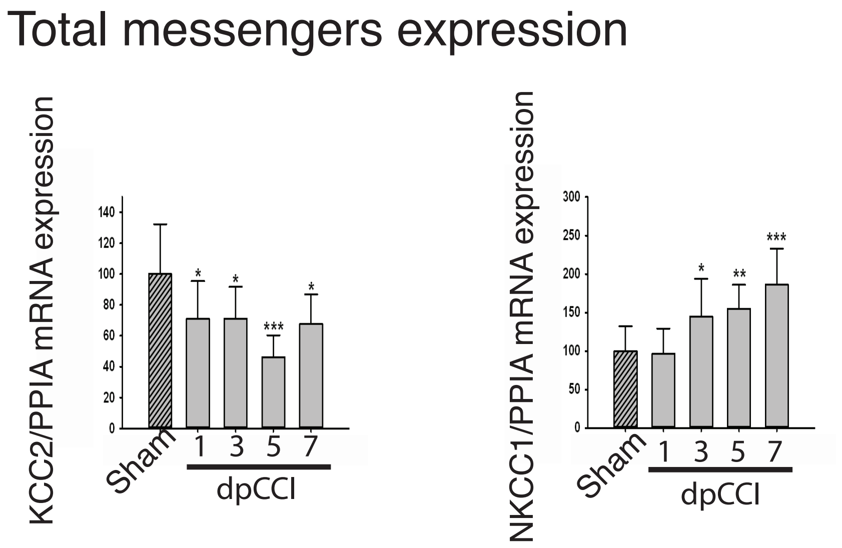

A) Relative KCC2 and NKCC1 mRNA expression is normalized to cyclophilin A gene (PPIA) at different time after trauma during the first post-traumatic week (n=10 per condition). One-way Anova test is performed and expressed as following *p<0,05; **p<0,01; ***p<0,001.
